# Sudden Cardiac Arrest in a Patient With COVID-19 as a Result of Severe Hyperkalemia After Administration of Succinylcholine Chloride for Reintubation. A Case Report

**DOI:** 10.3389/fmed.2022.843282

**Published:** 2022-05-11

**Authors:** Mateusz Putowski, Tomasz Drygalski, Andrzej Morajda, Jarosław Woroń, Tomasz Sanak, Jerzy Wordliczek

**Affiliations:** ^1^Center for Innovative Medical Education, Jagiellonian University Medical College, Cracow, Poland; ^2^Department of Anesthesiology and Intensive Care, University Hospital in Krakow, Krakow, Poland; ^3^Department of Clinical Pharmacology, The Chair of Pharmacology, Faculty of Medicine, Jagiellonian University Collegium Medicum, Krakow, Poland; ^4^Department of Intensive Interdisciplinary Therapy, Jagiellonian University Collegium Medicum, Krakow, Poland

**Keywords:** COVID-19, hyperkalemia, sudden cardiac arrest, rocuronium, succinylcholine

## Abstract

**Background:**

We present a case study of a man with coronavirus disease 2019 (COVID-19) who developed cardiac arrest as a result of hyperkalemia following administration of chlororsuccinylcholine during endotracheal intubation.

**Case Summary:**

A patient with a severe course of COVID-19, hospitalized in the Intensive Care Unit, underwent reintubation on day 16. The applied scheme was rapid sequence induction and intubation with administration of chlororsuccinylcholine. Immediately after intubation, there was a sudden cardiac arrest due to hyperkalemia (cK + 10.2 meq/L). Treatment was initiated as per guidelines, which resulted in a return to spontaneous circulation after 6 min.

**Conclusion:**

Chlorsucynylcholine may cause life-threatening hyperkalemia. We recommend using rocuronium as a neuromuscular blocking agent in critically ill COVID-19 patients due to its more optimal safety profile.

## Introduction

Succinylcholine chloride is an agent commonly used to facilitate endotracheal intubation. It also remains a major drug in rapid sequence induction and intubation in many countries (1–3). Nevertheless, the British Guidelines for the management of tracheal intubation in critical ill adults recommend rocuronium as the first-line muscle relaxant for endotracheal intubation in critical condition (4). However, it is succinylcholine, as a depolarizing agent with a fast (40–60 s) and short (6–10 min) duration of action, that is more eagerly chosen in emergency conditions due to its pharmacokinetic profile compared to rocuronium, whose duration of action is about 37–72 min depending on dose (5, 6). However, it should be remembered that this drug may cause side effects such as malignant hyperthermia, rhabdomyolysis, or hyperkalemia. There are also more and more reports of sudden cardiac arrest (SCA) following the supply of succinylcholine (7, 8). Our case report is further evidence of serious complications following the administration of this drug for endotracheal intubation in a patient with a severe course of coronavirus disease 2019 (COVID-19).

## Case Report

**FIGURE 1:**
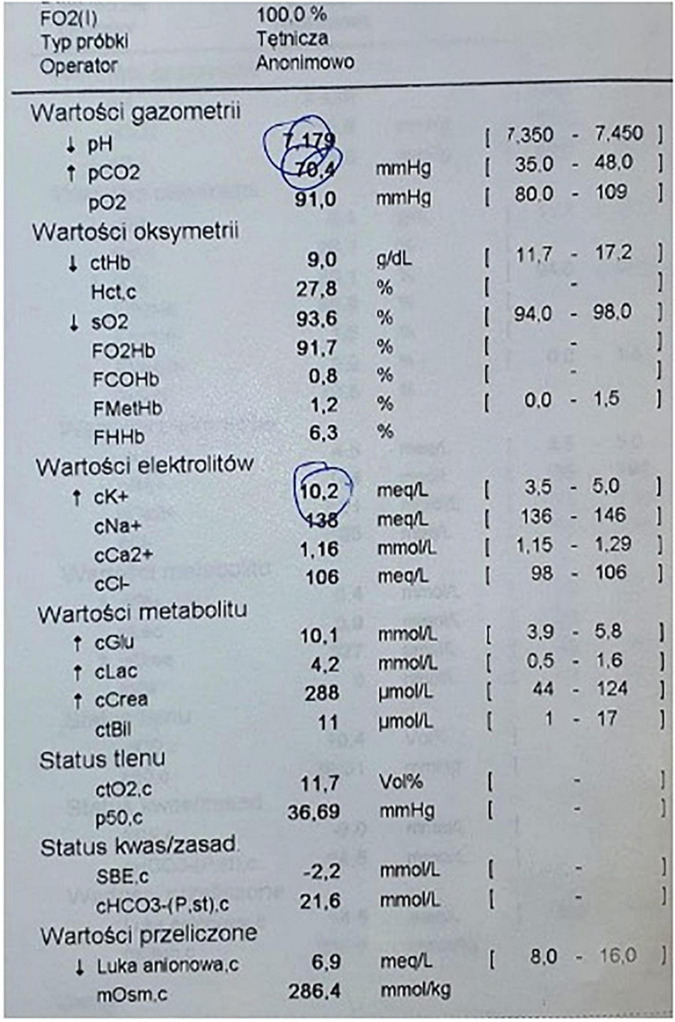
Arterial blood gas obtained during cardiopulmonary resuscitation, time of the result 4:11 PM.

**FIGURE 2:**
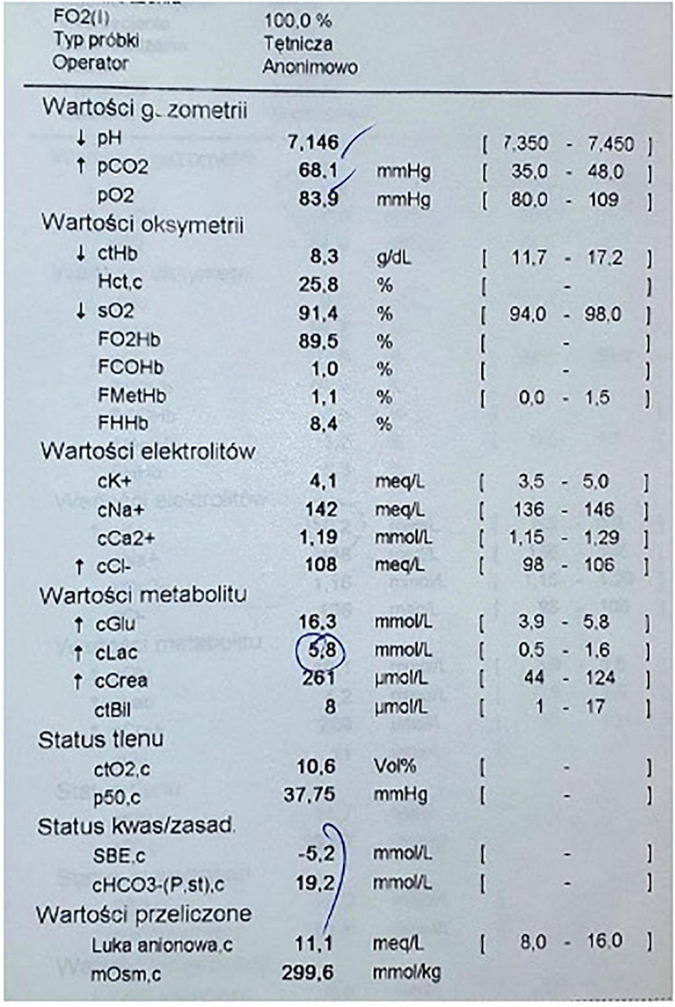
Arterial blood gas obtained after recovery of spontaneous circulation, time of the result 4:22 PM.

A 57-year-old patient with COVID-19 was admitted to the Anesthesiology and Intensive Care Unit after being transferred from another hospital for further treatment due to increasing respiratory failure. Patient transported by medical transport team was intubated, in critical general condition, in analgosedation, mechanically ventilated in the SIMV (*synchronized intermittent mandatory ventilation*) mode with Fi0_2_ (*fraction of inspired oxygen*) 0.5 at PEEP (*positive end-expiratory pressure*) 14 cmH_2_O. After admission, multidisciplinary and integrated treatment, empiric antibiotic therapy were implemented in accordance with the local protocol, taking into account the drug-susceptibility phenotypes of potential bacterial pathogens, antithrombotic and antiedematous prophylaxis was applied, and water-electrolyte imbalance was corrected. Mechanical ventilation mode was changed to VC/AC (Volume Control/Assist Control) with Fi0_2_ 0.4 reduction and PEEP 15 cmH_2_O. During hospitalization, the patient was placed in the prone position several times in order to improve ventilation parameters. On day 8 from admission, renal replacement therapy was administered in the mode of continuous venous-venous hemodiafiltration (CVVHDF) with an Oxiris and CytoSorb filter. On day 16, the ventilation mode was changed to pressure support ventilation and the settings were modified based on arterial blood gas test. Due to the long time since changing the previous tracheal tube, a decision was also made to reintubate the patient. Mouth opening of about 3 cm, Mallampati score III and arterial blood gas results from 2 h ago (pH 7.43, *P*O_2_ 75 mmHg, kPa, *P*CO_2_ 35 mmHg, cK + 4.5 meq/L, cLac 0.9 mmol/L, and SBE 0.0 mmol/L) were assessed. Immediately prior to intubation, the patient was under continuous infusion of noradrenaline (Levonor) and dexmedetomidine (Dexdor) and CVVHDF renal replacement therapy. The classic RSI regimen was used: fentanyl (1.5 μg kg^–1^), propofol (1 mg kg^–1^), and succinylcholine (Chlorsuccillin, 200 mg, Bausch Health Ireland Limited) at a dose of 1 mg kg^–1^. After that, the tracheal tube was replaced using a Bougie type guide in about 20 s. Immediately after replacement of the endotracheal tube, cardiac arrhythmias in the form of bradycardia 50 bpm with wide QRS complexes and a sudden drop in invasive blood pressure were noticed on the cardiomonitor. The carotid pulse was assessed, and upon finding its absence, cardiopulmonary resuscitation was started immediately and adrenaline 1 mg was administered iv due to the non-shockable rhythm. Due to the unexpected onset of SCA in this situation, it was suspected that hyperkalemia may have occured after administration of chlorsuccinylcholine. In the next minute of CPR, arterial blood gas test was obtained and acidosis was found with severe hyperkalemia (cK + 10.2 meq/L; Figure 1). 10 ml of 10% calcium chloride were administered immediately, insulin (10U) with glucose (25 *g*) was infused and 60 meq of Natrium bicarbonicum 8.4% was administered. Subsequent evaluation of the rhythm, however, did not return the spontaneous circulation, and the rhythm was still non-shockable. At approximately 6 min of CPR, the spontaneous circulation was restored and the heart rate normalized to approximately 70 bpm with narrow QRS complexes. Arterial blood gas was again taken, showing a decrease in potassium ions to 4.1 meq/L (Figure 2). After this incident, the patient stayed in our ward for 22 days. He was discharged in a stable state, conscious in logical verbal contact, from another ward in order to continue the treatment. The co-author and supervisor of this publication is the Head Physician of our Department, who gave his written permission to describe this case.

## Discussion

The presented case study is further evidence of the risk of severe hyperkalemia following the administration of succinylcholine. In a review by Hovgaard et al. on suxamethonium-induced hyperkalemia and the Cochrane review regarding rocuronium versus sucinylcholine in the rapid intubation sequence not only explained the causes of the succamethonium-induced potassium upsurge, but also identified a group of patients at particular risk of this complication. Patients with severe burns, trauma, immobilized and prolonged critical illness have been shown to be at high risk of developing life-threatening hyperkalemia following administration of this drug (5, 9). It cannot be ruled out that the SARS-CoV-2 virus infection itself is another risk factor for this complication. The coronavirus infection itself can change the pharmacokinetic parameters of many drugs (10). Electrolyte disorders particularly potassium abnormalities have been repeatedly reported as common clinical manifestations of COVID-19. SARS-CoV-2 cell entry through angiotensin-converting enzyme 2 may enhance the activity of renin–angiotensin–aldosterone system classical axis and further leading to over production of aldosterone. Aldosterone is capable of enhancing the activity of epithelial sodium channels and resulting in potassium loss from epithelial cells. Damage to the renal tubules caused by ischemia and nephrotoxic effects caused by inflammation in the body is also not without significance for the disturbance of potassium homeostasis. The SARS-CoV-2 virus also influences the regulatory mechanism of the renin-angiotensin-aldosterone system. SARS-CoV-2 can lead to both decreases and increases in serum potassium levels (11).

Hyperkalemia occurs in a small subset of patients after succinylcholine (SCh) administration and can be severe and fatal. In a review of cases and the pathophysiology of succinylcholine hyperkalemia, Gerald et al. describes 2 mechanisms that lead to the disorder: upregulation of acetylcholine receptors and rhabdomyolysis. Upregulation is caused by a change in the subunit type of acetylcholine receptors and by an increase in their density as they spread over the muscle surface outside the motor endplate area. Causes of upregulation include burns, severe muscle trauma, upper or lower motor neuron denervation (e.g., stroke or spinal cord injury, respectively), and prolonged ICU care (bed rest, steroids, prolonged neuromuscular blockade). Both succinylcholine and acetylcholine are agonists of the acetylcholine receptor. This channel-related agonist-triggered potassium release is magnified by the number of involved muscles (12).

Unfortunately, the global disease pandemic caused by the SARS-CoV2 virus has resulted in a significant increase in people hospitalized in Intensive Care Units due to severe respiratory failure. The constant deterioration of ventilation parameters results in an urgent need for endotracheal intubation and the implementation of assisted or mechanical ventilation. Post-intubation cardiac arrest is a rare complication of intubation, but it should be noted that the number of such cases increased 1.5 times during the COVID-19 pandemic (13).

When choosing a drug regimen for RSI, one should take into account the risk of serious complications. An alternative to succinylcholine is rocuronium administered at a dose (up to RSI) of 1.2 mg/kg bw. Its main advantage is the lack of potassium ion surge, which eliminates the occurrence of hyperkalemia, but the duration of action is much longer, which may be a potential problem. There is indeed an antagonist for rocuronium (i.e., sugammadex), however, large doses of this drug, i.e., 16 mg/kg bw are needed to reverse the neuromuscular blockage, and the time to fully reverse the muscle blockage may take as long as 3 min (14, 15). Rocuronium also has a longer apneic window without desaturating oxygen level (“Safe Apnea Time”) which allows for a change in airway management in the event of in the event unanticipated complications arise in obese patients (16).

Blanié et al. studies have shown that the risk of hyperkalemia after the administration of succinylcholine is also strongly correlated with the length of stay in the ICU. After 16 days of hospitalization, the risk of this complication is very high (17).

Sigurdsson et al. describe a similar case of cardiac arrest after administration of succinylcholine in a patient with COVID-19, but the patient showed hypoxemia and respiratory acidosis before intubation (pH 7.28, P O2 63.0 mmHg, Pco2 67.5 mmHg, and potassium 4.7 meq/L). About 60 s after drug administration, circulatory arrest occurred in a defibrillation rhythm (wide complex polymorphic ventricular tachycardia), and the hyperkalemia recorded by the authors was 6.4 meq/L of potassium (7). The patient described in our report had no acid-base imbalance prior to intubation and additionally remained on venous-venous hemodiafiltration (CVVHDF). After administration of the drug, there was also a typical hyperkalemic arrhythmia in the form of broad QRS bradycardia, and the recorded potassium level was 10.2 meq/L (18). Another factor that should be taken into account in the case described by Sigurdsson et al. is hypercapnia. It is important to realize that during an apnea following administration of a muscle relaxant, severe acidosis may occur due to an increase in PaCO2, leading to cardiac arrest (16).

In our opinion, the supply of chlorsuccinylcholine was the main cause of cardiac arrest in this patient. We recommend that prior to selecting muscle relaxants for endotracheal intubation in the intensive care unit, one should assess not only serum potassium levels, but also hospitalization time, and use rocuronium as a neuromuscular blocking agent in critically ill patients with COVID-19 due to its more optimal safety profile.

## Data Availability Statement

The original contributions presented in the study are included in the article/supplementary material, further inquiries can be directed to the corresponding author.

## Author Contributions

All authors listed have made a substantial, direct, and intellectual contribution to the work, and approved it for publication.

## Conflict of Interest

The authors declare that the research was conducted in the absence of any commercial or financial relationships that could be construed as a potential conflict of interest.

## Publisher’s Note

All claims expressed in this article are solely those of the authors and do not necessarily represent those of their affiliated organizations, or those of the publisher, the editors and the reviewers. Any product that may be evaluated in this article, or claim that may be made by its manufacturer, is not guaranteed or endorsed by the publisher.
